# ‘Too old to test?’: A life course approach to HIV-related risk and self-testing among midlife-older adults in Malawi

**DOI:** 10.1186/s12889-021-10573-7

**Published:** 2021-04-03

**Authors:** Cheryl Johnson, Moses Kumwenda, Jamilah Meghji, Augustine T. Choko, Mackwellings Phiri, Karin Hatzold, Rachel Baggaley, Miriam Taegtmeyer, Fern Terris-Prestholt, Nicola Desmond, Elizabeth L. Corbett

**Affiliations:** 1 Global of HIV, Hepatitis and STIs Programmes, World Health Organization, 20 Ave Appia, 1211 Geneva, Switzerland; 2 Department of Clinical Research and Infectious Disease, London School of Hygiene and Tropical Medicine, London, UK; 3 Malawi Liverpool Wellcome Trust, HIV/TB Group, Blantyre, Malawi; 4 Helse Nord TB Initiative, College of Medicine, Blantyre, Malawi; 5 Department of Clinical Sciences, Liverpool School of Tropical Medicine, Liverpool, UK; 6 Population Services International, Johannesburg, South Africa; 7 Tropical Infectious Diseases Unit, Royal Liverpool University Hospital, Liverpool, UK; 8 Department of Public Health and Policy, London School of Hygiene and Tropical Medicine, London, UK

**Keywords:** Life-course theory, Age stratification, Socioemotional selectivity, HIV/AIDS, HIV self-test

## Abstract

**Background:**

Despite the aging HIV epidemic, increasing age can be associated with hesitancy to test. Addressing this gap is a critical policy concern and highlights the urgent need to identify the underlying factors, to improve knowledge of HIV-related risks as well as uptake of HIV testing and prevention services, in midlife-older adults.

**Methods:**

We conducted five focus group discussions and 12 in-depth interviews between April 2013 and November 2016 among rural and urban Malawian midlife-older (*≥*30 years) men and women. Using a life-course theoretical framework we explored how age is enacted socially and its implications on HIV testing and sexual risk behaviours. We also explore the potential for HIV self-testing (HIVST) to be part of a broader strategy for engaging midlife-older adults in HIV testing, prevention and care. Thematic analysis was used to identify recurrent themes and variations.

**Results:**

Midlife-older adults (30–74 years of age) associated their age with respectability and identified HIV as “a disease of youth” that would not affect them, with age protecting them against infidelity and sexual risk-taking. HIV testing was felt to be stigmatizing, challenging age norms, threatening social status, and implying “lack of wisdom”. These norms drove self-testing preferences at home or other locations deemed age and gender appropriate. Awareness of the potential for long-standing undiagnosed HIV to be carried forward from past relationships was minimal, as was understanding of treatment-as-prevention. These norms led to HIV testing being perceived as a threat to status by older adults, contributing to low levels of recent HIV testing compared to younger adults.

**Conclusions:**

Characteristics associated with age-gender norms and social position encourage self-testing but drive poor HIV-risk perception and unacceptability of conventional HIV testing in midlife-older adults. There is an urgent need to provide targeted messages and services more appropriate to midlife-older adults in sub-Saharan Africa. HIVST which has often been highlighted as a tool for reaching young people, may be a valuable tool for engaging midlife-older age groups who may not otherwise test.

**Supplementary Information:**

The online version contains supplementary material available at 10.1186/s12889-021-10573-7.

## Background

Aging among people living with HIV (PLHIV) has attracted significant interest in recent years. While treatment scale-up has resulted in better quality and longer life, numbers of new infections are not declining, and indeed are increasing in older (> 50 years) adults [1]. In sub-Saharan Africa, HIV prevalence among older men and undiagnosed infections are on the rise: in 2015, 50% of new HIV infections among men in sub-Saharan Africa were in 30–49 year olds [2, 3]. With the median age of PLHIV increasing every year [2], by 2030 approximately 73% of PLHIV will be over 50 years [4] with the vast majority in sub-Saharan Africa.

Low levels of recent HIV testing in midlife-older populations, generally ≥30 years of age, stand in contrast with the changing epidemic in southern Africa. In east and southern Africa – where 85% of all PLHIV are now aware of their status [1] – many midlife-older adults, especially men, have still never tested or not tested recently [5]. Late diagnosis and initiation of treatment is common among midlife-older men, who prescribe to traditional views of masculinity and age which view accessing HIV services, including testing, as a threat to their position in community and family life [6, 7]. This trend among midlife-older men may also contribute to new infections in younger women in southern Africa where intergenerational relationships have been considered a key driver of the HIV epidemic [8]. For midlife-older women, some of whom no longer need antenatal or family planning services, access to and uptake of HIV testing declines considerably [9]. Declining concerns about unwanted pregnancy, together with minimal public awareness of changes in the demographics of the HIV epidemic [10, 11], may lead to greater willingness to engage in condomless sex with existing or new partners of unknown HIV status [12–14].

Despite increasing HIV prevalence, risk perception and perceived susceptibility to HIV is believed to be low in midlife-older age groups [9, 15, 16]. Age-targeted health education and behaviour change campaigns in southern Africa remain focused on adolescents and young people (15–24 years) and have yet to be adapted for other age groups. As a result, in urban Malawi, only 42.8 and 66.7% of over-40-year-old men and women participated in the first year of community-based HIV self-testing (HIVST) distribution in 2012–13 and had an HIV prevalence of 22.5%. In contrast, during this same period, 89.3 and 100% of adolescent boys and girls aged 16–19 years participated with a lower HIV prevalence of 2.5% [17]. Since then, Malawi has scaled-up HIVST and youth continue to be a priority [18].

Poor knowledge of their HIV risk and hesitancy to access HIV testing in the context of high prevalence and incidence among midlife-older men and women undermine global efforts to achieve and maintain low HIV incidence by 2030. Addressing this gap is a critical policy concern and highlights the urgent need to identify the underlying factors, to improve knowledge of HIV-related risks as well as uptake of HIV testing and prevention services, in midlife-older adults.

### Theoretical framework

The life-course comprises the set of socially defined events and roles individuals enact over time [19]. Within the life-course, in addition to chronological age, aging is socially and historically constructed by different life events, experiences, and social expectations which shape individual behaviours, perceptions and attitudes [20, 21]. These age constructs inform how individuals enact and cultures express “age” through different life stages, such as adulthood, midlife and elderhood, and their meaning in society [20, 22–24]. Within these constructs culturally-dominant ideals of achievement, respectability and status contribute to shared age-related identities [25]. Diversity of experiences informed by gender, power, class, place and time also influence and define multiple and continuous expressions of age within and across cultures [26–28].

Studies in southern Africa have begun to apply life-course approaches to understanding HIV among older adults [9, 16, 29–35]. Findings have highlighted how age norms can make accessing HIV prevention, testing and care challenging. This is often due to concerns about loss of respect and age-related stigma and discrimination reflecting experiences from earlier times when treatment was not widely available. Age norms can also be gendered by traditional masculinities reducing HIV testing and ART initiation among older men [7], or increased household and community responsibilities becoming a barrier to health services, including HIV testing, among older women [12, 22, 29].

The vast majority of studies continue to focus on aging as it relates to trends in HIV epidemiology, risk factors and issues following an HIV-positive diagnosis [10, 11, 15, 29, 36]. Framing the discussion around the life-course [19], while also considering age and gender [20, 25], contributes to the literature by examining the gendered construction of ‘respectable’ midlife-older age adult life in southern Africa, and how it influences perceptions of HIV-related risk and both conventional HIV testing and self-testing in urban and rural Malawi.

Here we explore perceptions of HIV risk and HIV testing among midlife-older men and women living in rural and urban Malawi following the introduction of HIVST. Drawing on theories of life-course [20, 21] and performance of self in society [24], we use qualitative methods to understand how social age is enacted and implications for HIV testing and sexual risk behaviours among midlife-older Malawians. We also explore the potential for HIVST to be part of a broader strategy for engaging midlife-older adults in HIV testing, prevention and care.

## Methods

This qualitative study was nested in two cluster-randomised trials of community-based HIVST, one (ISCTN02004005) in three high-density townships of Ndirande, Likhubula and Chilomoni in urban Blantyre and one (NCT02718274) in Manyenje and Nkoka villages in rural Blantyre, Malawi. Results, including uptake of HIVST by age and sex have been described elsewhere [17, 18, 37]. Briefly, volunteers from intervention communities were trained as community-based distributors (CBDs) responsible for providing HIVST to their neighbours [17, 38]. Distributors provided residents with pre-test information, including a demonstration on how to use an oral HIVST kit (OraQuick HIV Self-Test, OraSure Technologies, LLC, Bethlehem, PA, USA). Community members could self-test with a CBD or take a kit home to test later. All self-testers were informed by CBDs that they needed to confirm reactive results and where to access treatment. Disclosure of self-test results was not required, though many shared results with CBDs [17, 18, 37].

Five focus group discussions (FGDs) (*n* = 48) and 12 in-depth interviews (IDIs) were conducted between April 2013 and November 2016 in five HIVST intervention areas. Community residents were eligible for FGDs if 35 years or older, and IDIs if over 30 years. FGD and IDI participants were recruited prospectively, by liaising with CBDs. Participants were only engaged once for approximately 1 h and were provided transport and refreshments. FGDs included: two with both men and women cluster residents, two with women only, and one with community distributors only. FGDs ranged from 8 to 12 participants, including 48 participants in total. IDIs were limited to community members who had self-tested (Table 1).

**Table 1:** Demographic characteristics of community residents and distributors

	FGD (***n*** = 4)	CBD FGD (***n*** = 1)	IDI (***n*** = 12)
	*# of participants, n = 37*	*# of participants,* *n = 11*	*# of participants,* *n = 12*
**Sex**			
Male	9	6	6
Female	28	5	6
**Age (years)**			
Median age	39 years (range:35–74)	33 years (range: 24–61)	35 years (range: 31–64)
< 35	0	7	5
35–49	34	3	5
50+	2	1	2
Unknown	1	0	0
**Education**			
Adult literacy	1	0	0
Primary incomplete or complete	15	0	7
Secondary school complete	0	9	2
Some secondary education	21	2	3
**Marital Status**			
Married or living as married	23	*	10
Widowed, separated or divorced	14	*	1
Never married	0	*	1
**HIV status**			
HIV positive	9	*	2
HIV negative	24	*	10
Unknown	4	*	0
**Ever tested**			
Yes	20	*	8
No	5	*	4
Unknown	12	*	0
**Self-tested**			
Yes	32	*	12
No	5	*	0

*FGD* Focus group discussion, *CBD* Community-based distributor, *IDI* In-depth Interview

Community FGDs and IDIs explored personal experiences and community perceptions, including aspects of the life-course, such as: age and gender norms, social positions, as well as knowledge of HIV and treatment, risk perception, stigma, social harm, self-testing, relationships with distributors, and perceptions of barriers to testing. The distributor FGD explored their experiences offering HIVST to middle-aged and older community members, and perceived barriers to testing. Topic and interview guides are publicly available on the London School of Hygiene and Tropical Medicine (LSHTM) website [39]. All participants in IDIs and FGDs were assured that information shared was confidential and identifiable information would not be shared. FGD participants were requested to maintain confidentiality related to discussion. All participants were anonymized and identifying information was de-linked using a unique study code prior to review and analysis. Unique participant study codes were only made accessible to the research team.

FGDs and IDIs were conducted in the local language (Chichewa) by three male field workers experienced in qualitative methods (MK, MP, and LK) and audio recorded. One field worker led each FGD and IDI. All field workers were trained, participated in development of interview guides and had extensive experience working in the community as qualitative researchers and were native speakers. All recordings were transcribed into Chichewa and then translated into English by trained personnel. Electronic data was stored in password protected servers (RedCap) and password protected computers. Paper-based data was kept in a locked cabinet which was only accessible to the research team.

Thematic content and framework analysis were conducted on FGD and IDI transcripts using a common coding framework developed under the STAR Initiative for data sorting and indexing. Transcripts were first read (CJ, JM, MK) and grouped by themes and then triangulated across participants by age, gender, HIV status and setting (urban or rural). Transcripts were read and re-read in English and Chichewa, then coded themes were jointly re-reviewed to ensure consistency (CJ, MK). Following which, themes were extracted and used to refine the framework iteratively and inform the analysis that was software assisted by NVIVO 12 (QSR International Pty Ltd.). Study findings, from Self-Testing Africa (STAR) and HitTB, were then disseminated to local communities and the ministry of health. Individual participants, however, did not review results.

Ethical approval was obtained from LSHTM, and Malawi College of Medicine and Research and Ethics Committee (COMREC) (P011/10/1020). According to COMREC approved procedures, all participants were informed about the study and goals to evaluate HIVST. All literate participants provided written informed consent and all illiterate participants provided verbal witnessed informed consent and a thumb print. The study and presentation of results followed COREQ guidelines.

## Results

Community residents participating in the study were aged 30–74 years. Additional characteristics and key FGDs and IDIs quotes are summarised in Tables 1 and 2 in addition to select quotes which highlight key themes. Data presented, and findings were consistent. Supplementary information includes full summary of participant quotes (S1).

**Table 2:** Select community resident and distributor quotes on social position, HIV-related risks and HIV testing from focus group discussions and in-depth interviews across the life course

Key themes	#	Participant quote	Participant characteristics
**Defining age and midlife-older adulthood**			
Age as behaviour	1	If your behaviour is not good … even a child thinks you are also a child. While if a little child gives himself respect, some people also respect him as if he is older.	Community resident, 35–49 years, urban, focus group discussion (FGD)
	2	The one who is looked at as an old person, is the one who follows the advice that he has been given, because when an old person is told something he follows the rules. The one whom we consider as a young person, is the one who doesn’t follow the advice that people give him.	Community resident, 35–49 years, urban, FGD
	3	We know a young person [by] their behaviour … it’s like a prodigal life. They drink a lot, smoke chamba, fornication is becoming rampant in the young ones.	Community resident, 35–49 years, urban, FGD
Social expectations and standing			
HIV is a ‘disease of youth’	4	How could an elderly person like this be found with a disease like this? It should have happened to the youth because they are the ones who ‘run faster’ (are more active sexually).	Community resident, 35–49 years, urban, in-depth interview (IDI)
	5	[Older people] think there is no reason to go for testing because in their time there was no HIV. HIV is a disease for people who were born after the year 1985. They think the disease is not part of them as they were born and grew up before the disease was discovered.	Community-based distributor (CBD), 35–49 years, urban, FGD
	6	Most people think AIDS is a disease for the youth, because old people are the ones who give advice. If they are giving advice to the youth and then they should also contract the virus, it becomes surprising. It makes people ask a lot of questions.	Community resident, 35–49 years, urban, FGD
	7	When an old person is looking at a young person, he thinks that a young person has [HIV] in his body. But when a young person is looking at an old person he is 100% sure that this old person does not have any [HIV] in his body.	Community resident, 35–49 years, urban, FGD
Risk perceptions in relation to modes of HIV transmission			
Infidelity and trust	8	No I don’t have concerns, even though I am not in marriage but I have a partner and I have trust in her.	Community resident, < 35 years, rural, IDI
	9	You can say this is my wife and we are loving each other without knowing that you are thinking differently.	Community resident, 50+ years, rural, IDI
	10	*[Interviewer: You had any perception of risk of HIV then before found positive with self-testing?]* Yes, because my husband had a relationship with a woman who was HIV positive and she was on ARVs … My husband doesn’t stop his immoral behaviour.	Community resident, 35–49 years, rural, IDI
Non-sexual modes of transmission	11	We explain to them that one can contract the virus through different ways. It might be that you helped a certain person, or maybe you used something sharp, and from nowhere you can easily contract the virus. Because of that, they say ‘I think that you are explaining well’ and you will find that they get tested.	CBD, < 35 years, urban, FGD
	12	… I always have fear with barbershops that cant we get HIV? I just think that because everyone use the same [shaving] machine.	Community resident, 50+ years, rural, FGD
	13	… I used bath soaps, so maybe through that I can have a concern [HIV risk].	Community resident, 50+ years, rural, FGD
Consequences of HIV testing and diagnosis in later life			
Social and self-stigma	14	If you go for HIV-testing people say you doubt yourself. They talk a lot saying there is something making you go for testing. They don’t look at it as if it’s just your decision, or it is because you listened to the counselling, or that you wish yourself a better future. They think that maybe you have been sleeping around or maybe you are getting sick.	Community resident, 35–49 years, urban, FGD
	15	Some older people ask us what will happen if they are found with the virus - will they receive the drugs right here at home or from the hospital? They say some people feel ashamed to go to the hospital and receive drugs, as there will be a queue for such things.	Urban CBD, < 35 years, urban, FGD
	16	If an old person has been found with the virus, people tend to wonder saying “aah how come?” because it’s like a young person is the one who is very active in sexual activities. So how has this old man contracted the virus? We look at those old people who contract HIV as if they lack wisdom.	Community resident, 35–49 years, urban, FGD
Fatalism	17	Some people say they are already dead when they test HIV positive, instead of start to receive ARVs.	Community resident, 50+ years, rural, IDI
	18	Some older people say ‘I have already grown up – what is remaining here is just dying. Why should I go to test? Even if they will mend me, what will that do for me?’	Community resident, 50+ years, rural, FGD
Condom use and abstinence	19	Since that incident happened [both diagnosed with HIV], the community health worker came and gave us condoms. That’s what we are using now. We are using condoms, apart from that we usually having sex once per week or 2 weeks.	Community resident, 35–49 years, rural, IDI
	20	Yes, we use [condoms] … We will continue. As for this unborn baby, [I am protecting it through] the treatment I am receiving.	Community resident, < 35 years, rural, IDI
Perceptions and experiences with HIV self-testing			
Ease of use and time saving	21	Yes, I would recommend because the procedure is simple.	Community resident, < 35 years, rural, FGD
	22	It was very simple to self-test, I just followed the instructions and managed to test myself …. I would recommend self-testing because we save time instead of going to HTC [HIV testing and counselling] we do it ourselves at home. When you think about time and cost, is better to use self-testing because you will do it while at home. Self-test and you don’t waste your time, while at HTC you need to travel and spend money for transport and you will be tested by the doctor.	Community resident, < 35 years, rural, IDI
	23	[Self-testing] at home - you can do that within 15 min while you are doing other things at home, while testing at a facility it can take you over an hour.	Community resident, < 35 years, rural, IDI
	24	Aah, I don’t see any problems. I think there are only benefits because some people are not comfortable to go to the health facility for testing. So it is easier for them to use this method and know their status … Well, [when testing HIV-positive with self-test], I just accepted and admitted it … If I live in denial and be anxious it won’t solve anything. I had to accept and follow the counselling.	Community resident, < 35 years, rural, IDI
	25	The test kit is a very good thing because you are able to read the results yourself instantly. I believed the [positive] results …. There is benefit because it [self-testing] will bring trust and love to each other.	Community resident, 35–49 years, rural, IDI
Support during self-testing	26	The counselling regarding the kit itself would be to highlight how the apparatus works or how we can use it. After knowing how it works, then we would be able to use it. The only assistance I would want is advice regarding how to properly use the kit.	Community resident, 35–49 years, rural, IDI
	27	[Without guidance and supervision] it [would] be difficult because you don’t even know how to open the pack. For other people they can be easily to understand, while others it may be difficult for them, so to others might bring confusion.	Community resident, 50+ years, rural, FGD
	28	Old people prefer different things. Those who have reached 45 to 70 years are the ones who test in our presence so that we should help them in reading the results, and so you can explain the instructions to them properly. But people who are 28 to 40 years like to test by themselves because they know that may be their behaviour was not right at a certain time, and they know it wouldn’t be a problem to go to the hospital themselves.	CBD, 35–49 years, urban, FGD
Partner self-testing experiences and perspectives			
Family and couples counselling	29	The best counselling should be provided as a couple, because they will remind each other if one has forgotten.	Community resident, 50+ years, rural, IDI
	30	Counselling given to a family as a whole is good because it gives an opportunity for everyone to hear for himself … Especially [for] me and my wife	Community resident, 35–49 years, rural, IDI
Give kit to partner	31	Yes, I would be very glad because me and my wife are one. So if that could be the arrangement I believe she would be very glad to, because from the very beginning she was the one who was encouraging me to go for blood testing. With this kit, my wife would also be able to test herself.	Community resident, 35–49 years, rural, IDI
	32	I found myself to be HIV positive together with my husband. [Before] we had plans to go for testing, so we took self-testing together as an advantage to us, [and] we accepted the results … There is benefit because it [self-testing] will bring trust and love to each other.	Community resident, 35–49 years, rural, IDI
Preferences for future distribution of self-test kits			
Expectations of CBDs	33	Old people are stubborn to hear any advice from children. They don’t believe these children. They look at themselves as old people who have more wisdom. So, if a young counsellor goes to such a person, will they listen to him?	Community resident, 35–49 years, urban, FGD
	34	*[Interviewer: Who should distribute self-test kits in terms of age and sex?]* Anyone, as long as the person is trust worthy.	Community resident, 35–49 years, urban, IDI
	35	… Maybe your child will be conducting a test on me. I am an old person.	Community resident, 50+ years, urban, FGD

**Fig. 1:**
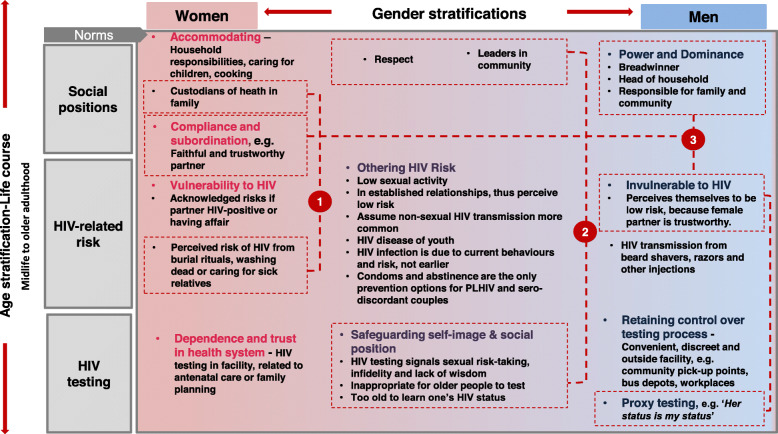
Example of perceptions of social positions, HIV-related risk and HIV testing by social age and gender among ‘respectable midlife-older adults in urban and rural Malawi. This figure is illustrative using three examples of age stratification-life course variation by gender among midlife-older adults in urban and rural Malawi: (1) Social positions of older women as custodians of health in the family is related to women’s representation of HIV risk, such as caring for sick relatives and burial rituals which are considered their responsibility and so, represent a ‘respectable’ risk that can be acknowledged openly; (2) Social positions of respect and being leaders in the community relates to midlife-older adults perceptions that testing is inappropriate for their age this HIV risks resulting in the “othering” of HIV-related risk behaviours as those which are contrary to characteristics of midlife-older adults; and (3) Social positions among older women as faithful and trustworthy relates to men’s views that they are low risk, and because of their lack of knowledge about HIV serodiscordancy among couples, they do not think they need HIV testing

The main themes, using a life-course framework, identified that there were stages of *elderhood*, with midlife-older age beginning around 30 to 35 years but mostly defined by respectable behaviour (e.g. low sexual activity, fidelity), attributes (e.g. wisdom) and life events (e.g. marriage, number of children) (see Fig. 1). Exploration of perceptions on HIV risk and HIV testing revealed views on respectable behaviour and age drove midlife-older adults to associate HIV with youth, but this reflected lack of knowledge and awareness of their own age-specific risks, including that HIV could have been acquired earlier and in previous relationships. The risk of sexual transmission at older ages was ignored: instead, there was a strong focus on non-sexual modes of transmission, implicitly considered more socially acceptable.

Because of their age, participants felt HIV testing could undermine their respectability, their roles and relationships in family and community life, and that health workers may stigmatize them if diagnosed with HIV. Learning one’s HIV-positive status later in life was considered stressful and deemed unhelpful, particularly as knowledge of the full benefits of ART and its role in preventing further transmission among those virally suppressed was very limited. As a result, midlife-older adults often considered conventional HIV testing unacceptable. Conversely, HIVST was highly preferable, for its convenience and privacy, especially through door-to-door distribution, though there were differences by age and sex.

Figure 1 illustrates emerging themes within a life course approach with variation by age and gender among ‘respectable midlife-older adults’ in urban and rural Malawi.

### Defining age and midlife-older adulthood

Participants drew from a range of norms and attributes to define elderhood and the start of midlife-older age in Malawi. While some focused on chronological age, with midlife-older age starting between 30 and 35 years, most defined this period by attributes and experiences [20], including the start of declining health or loss of strength, increased wisdom, respectable behaviour, major life events and increased responsibilities (e.g. marriage, parenthood). Because of this, many acknowledged that youth could be treated as “older”, based on marriage and through being compliant and respectable. Whereas those engaged in ‘bad’ or ‘unwise’ behaviours were considered “childish” at any age (Table 2, quotes (Q) 1–3).

The one who is looked at as an old person, is the one who follows the advice that he has been given, because when an old person is told something he follows the rules. The one whom we consider as a young person, is the one who doesn’t follow the advice that people give him. – Community resident, 35-49 years, urban, FGD

Participants voiced disapproval however when life events challenged life-course norms, such as older men leaving families and children, or pregnancy among young girls and older women.

### Midlife social positions and sexuality

Gender roles and responsibilities continued to follow traditional heteronormative dichotomies of men as head-of-household and women caring for the household and community. With age, both midlife-older adults, especially women, were expected to become more responsible, with diminishing sexual activity and infidelity. Being faithful and trustworthy was considered important for both genders, although most commonly described as a ‘mature’ female trait.

Older participants described themselves as ‘less sexually active’, more likely to be married, and less likely to have many partners. Indeed, having a ‘highly active sex drive’ in later life was considered socially unacceptable (Q4).

How could an elderly person like this be found with a disease like this? It should have happened to the youth because they are the ones who ‘run faster’ (are more active sexually). – Community resident, 35-49 years, urban, IDI

### Risk perceptions in relation to modes of HIV transmission

Men and women felt that only youth were affected by HIV, and midlife-older adults were at low risk (Q7). Sexual risk was discussed purely in the context of current behaviour, without acknowledging that a recent HIV diagnosis could reflect infection acquired years earlier.

When an old person is looking at a young person, he thinks that a young person has [HIV] in his body. But when a young person is looking at an old person he is 100% sure that this old person does not have any [HIV] in his body. – Community resident, 35-49 years, urban, FGD

Infidelity by oneself or one’s partner was an acknowledged risk for acquiring HIV within both urban and rural communities. Few participants however were willing to acknowledge this as a risk within their own relationship. Men spoke more frequently and openly about infidelity, including hinting of doubts about their partner, but still rated themselves as being at low risk. The exception was when a partner was known to be HIV-positive or was known to have had an affair with someone known to be HIV-positive (Q10).

[*Interviewer: You had any perception of risk of HIV then, before found positive with self-testing?]* Yes, because my husband had a relationship with a woman who was HIV positive and she was on ARVs … My husband doesn’t stop his immoral behaviour. – Community resident, 35-49 years, rural, IDI

While sexual transmission of HIV was acknowledged, midlife-older adults strongly emphasised non-sexual modes of transmission as a reason for older adults to worry about HIV and to consider HIV-testing. This reflected stated age norms of sexual inactivity, marriage, fidelity and respectability assigned to those considered older. Women in both rural and urban areas expressed concern about acquiring HIV through caring for the sick and bathing the dead. Routinely sharing items was another concern cited by both men and women, and including beard shavers, razors, soap and needles used for removing thorns. For CBDs, emphasising non-sexual routes of transmission provided a socially acceptable way of promoting HIVST amongst midlife-older adults, avoiding detailed discussions about sex which made participants less comfortable (Q11).

We explain to them that one can contract the virus through different ways. It might be that you helped a certain person, or maybe you used something sharp, and from nowhere you can easily contract the virus. Because of that, they say ‘I think that you are explaining well’ and you will find that they get tested. –CBD, < 35 years, urban, FGD

### Consequences of HIV testing and diagnosis in later life

Midlife-older adults considered themselves to be more subject to HIV stigma and at greater risk of losing social standing than younger people if diagnosed with HIV, or even if seen to be testing. They anticipated being considered ‘childish’, mocked and laughed at if diagnosed HIV-positive, and that their diagnosis would be interpreted as a ‘lack of wisdom’ and sexual impropriety (Q14–16).

We look at those old people who contract HIV as if they lack wisdom. – Community resident, 35-49 years, urban, FGD

The extent to which these concerns were justifiable, and from what age, however, was unclear: for instance, neither of the two married women in their 30s who disclosed that they were diagnosed with HIV through self-testing experienced any negative reactions (Q24–25).

Awareness of HIV was considered psychologically stressful, with some older adults considering themselves to be “already finished” with little to gain from learning their HIV status. ART was considered beneficial to health by all, although sometimes difficult to access. And there was little evidence of awareness of treatment-as-prevention, with newly diagnosed participants instead stating intent to use condoms or practice sexual abstinence with their spouse (Q17; Q19).

Some older people say ‘I have already grown up – what is remaining here is just dying. Why should I go to test? Even if they will mend [treat] me, what will that do for me?’ – Community resident, 50+ years, urban, FGD

Since that incident happened [both diagnosed with HIV], the community health worker came and gave us condoms. That’s what we are using now. We are using condoms, apart from that we usually having sex once per week or two weeks. – Community resident, 35-49 years, rural, IDI

In this context, testing for HIV was considered stigmatizing for older adults as there was widespread belief that wanting to test would be interpreted as evidence of recent infidelity or sexual risk-taking and that testing HIV-positive would only be harmful. The need for complete confidentiality was stressed for the act of testing, as well as the results, with caution expressed even for home-based or community programmes visible to family and neighbours even though participants recognised that those testing HIV-positive would inevitably lose all confidentiality as soon as they were seen to be attending their local ART clinic.

### Experiences and concerns relating to self-testing

Community-based HIVST, with support and guidance from a CBD and the option to give a kit to a partner, was considered to have many advantages for midlife-older adults, addressing their concerns by providing confidentiality and stigma, as well as convenience. Older participants desired more support compared to younger participants while self-testing, which was confirmed by CBDs (Q26–28).

Old people prefer different things. Those who have reached 45 to 70 years are the ones who test in our presence so that we should help them in reading the results, and so you can explain the instructions to them properly. – CBD, 35-49 years, urban, FGD

Being able to give an HIVST kit to a partner or self-test with a spouse, having decided and received information and counselling together, was considered advantageous by midlife-older adults (Q29–32).

I found myself to be HIV positive together with my husband. [Before] we had plans to go for testing, so we took self-testing together as an advantage to us, [and] we accepted the results … There is benefit because it [self-testing] will bring trust and love to each other. – Community resident, 35-49 years, rural, IDI

Neither gender, however, liked the idea of having a self-test kit imposed on them by their partner via “secondary distribution”, reflecting themes of HIV testing undermining social position, as well as questioning one’s elderhood by doubting their fidelity.

Concerns about risks posed to the community by HIVST were negligible for all age-groups, with anticipated benefits considered to outweigh harms. No social harm was reported by participants who all previously self-tested, including two women who disclosed that they were diagnosed with HIV through self-testing.

### Future service delivery preferences for self-testing

Many urban participants considered younger CBDs inappropriate for older community members, being unable to discuss personal issues and unlikely to be persuasive (Q33; Q35). However, older participants in rural settings prioritized trustworthiness over age or sex of distributors (Q34).

*[Interviewer: Who should distribute self-test kits in terms of age and sex?]* Anyone, as long as the person is trustworthy. – Community resident, 35-49 years, urban, IDI

This reinforced views that chronological age alone is not important, but that respectable behaviours and experience can also define what is considered “older”.

Acceptable alternatives to door-to-door distribution varied by age and gender, with women in their 30s suggesting outreach linked to antenatal and family-planning clinics, older women suggesting health facilities, and older men preferring fixed community collection points, workplaces or bus depots. These preferences aligned closely with perceptions of what was deemed age-gender appropriate.

Views on linkage post-HIVST did not appear to vary by age or gender, but some participants strongly preferred face-to-face post-test support. Following HIVST, having accompaniment from a relative or health worker or a referral slip (as in the study) was considered useful. Few other tools and approaches to support linkage were suggested.

## Discussion

Our study has applied the life course approach [20] to understand how midlife-older age (≥30 years) is defined and culturally expressed by Malawians not only as chronological age, but through social and gender norms and attributes which focus on what is considered respectable behaviour. These norms, coupled with limited awareness of the changes in the HIV epidemic, including high HIV prevalence in their age-group and treatment as prevention, result in low HIV-risk perception among older men and women in Malawi. Such findings support previous studies that have highlighted barriers and challenges with reaching older populations with HIV testing, prevention and treatment services in southern Africa [12, 22, 29].

Age norms defining midlife-older adulthood drove views that conventional HIV testing later in life is disreputable and unacceptable, implying infidelity, sexual risk-taking and a lack of wisdom that, if discovered, would threaten social position in family and community life. Socioemotional selectivity [40] was observed among older adults who highlighted concerns about stigmatising reactions, and that an HIV-positive diagnosis was too stressful and unhelpful. Awareness of the potential for long-standing undiagnosed HIV to be carried forward from past relationships was minimal, as was understanding of treatment-as-prevention. These norms led to HIV testing being perceived as a threat to status by older-adults, and likely drives the low levels of recent HIV testing in midlife-older adults [10, 15], as well as lower uptake of early introduction of HIVST in Malawi [17], compared to younger adults.

There is urgent need to communicate the changing epidemiology of HIV, and provide supportive HIV testing and prevention efforts, to midlife-older African adults, given their substantial underappreciation of personal risks, especially among + 35 year old men where HIV prevalence and new infections now exceeds younger age groups [1, 3]. Communicating the importance of condom use later in life, as well as how being on ART and maintaining viral suppression prevents transmission to sexual partners should be prioritized.

Older participants expressed marked preference for access to HIVST over other testing modalities, similar to younger age groups [17, 18, 41]. HIVST delivered at home, with an additional kit for a partner was generally acceptable among midlife-older adults, addressing concerns about being seen testing; though support in the testing process was needed.

Age and gender norms of what is ‘respectable’ for midlife and older adults emerged as key issues affecting HIV risk perceptions, knowledge and acceptability of HIV testing services. As previously reported [9, 16, 42, 43], the role and responsibility of women increased with age, including expectations of fidelity, being a caregiver and keeping the family healthy and HIV-negative. These preconceptions led many older men and women in established relationships to perceive their HIV risk to be low. Middle-aged and older adults strongly held the view that only young people could be affected because ‘mature adults’ were less sexually active, less susceptible to infidelity and had fewer partners.

This belief starkly contrasts with current epidemiological data [3, 31], but reflects cultural expectations of midlife and older Malawians, as well as gendered views of HIV, and early experiences and messages dating back to when HIV was first recognized in African societies [44] at which time HIV services and messages focused on reaching women and young people because of elevated risk. Such focus may have inadvertently reinforced views of low risk perception among older age groups, especially men, and undermined messages on the importance of testing and HIV prevention more broadly. These views, as well as limited knowledge of the full benefits of ART, emerged as key drivers of the concerns about potential stigma and social consequences of accessing HIV testing among older adults. The evidence on how to deliver effective HIV interventions and messages for older adults is limited [23, 45]. Further implementation research is needed to identify and scale-up messages and methods that effectively address sexual risk behaviour and HIV-related risks amongst midlife-older adults.

Our findings support HIVST as a useful tool for reaching midlife-older adults. The very same gender and social norms that drove low-risk perception and poor acceptability of HIV testing among mature adults, also drive preferences for self-testing. The feeling that testing at health facilities was too stigmatizing, costly and time consuming for older adults mirrored their stated preferences for self-testing because of its discretion and ease. While there was some variation, older adults preferred door-to-door HIVST, with an extra kit for a partner. Distribution by older providers, or those considered respectable, was often preferred. Acceptable HIVST kit access points varied by age and gender, defaulting to social positions and settings deemed gender appropriate (i.e. discreet collection points or workplace for older men and clinics for older women). Given the COVID-19 context, offering HIVST for midlife-older adults may also be increasingly advantageous to decongest health facilities and maintain essential services for those with potential risk factors that might lead to more serious disease in older populations.

Partner self-testing was viewed positively by all participants. Having an established social role and relationship was linked to this view, as well as having few concerns about harm in midlife-older adults. However, while individuals were inclined to give a self-test to a spouse, none stated they wanted to receive a kit from their partner. These findings further reinforce earlier reports showing both preference for giving a partner a kit [42], while receiving a kit is undesirable [46]. Considering continued reports of acceptability and high uptake of partner self-testing among men and women [47, 48], the lack of a stated preferences for receiving a kit from a partner is likely associated with socioemotional selectivity [27, 40] and avoiding undesirable relationship concerns, such as infidelity.

Future programmes should consider these preferences, and social and cultural norms of midlife-older adults, when planning HIVST, as well as broader HIV testing, implementation. Greater efforts however are still needed to reorient how age norms affect HIV risk and testing. Strategies including use of older providers and community workers who can provide support and instructions on how to self-test should be considered, as well as ambassadors who can align HIV prevention and testing behaviours with increasing respectability, health and responsibility among those who are older, and integrating HIV testing for older age groups as part of testing for non-communicable diseases [9, 16]. Information and counselling messages on the full benefits of treatment, particularly treatment as prevention are critical and may reduce some of the fear and stress of an HIV-positive diagnosis which inhibits testing later in life. Additional outreach strategies using traditional media, e.g. newspaper, radios, and village networks may be important as opposed to social media and other technologies being utilised to reach young people.

Our study has several limitations. First, although our study aimed to include a range of adults aged over 30 years, Malawi has a young population, with only 4 and 20%, respectively, aged over 50 and 35 years, as is reflected in our median age of participants of 41 years [49]. This does mean that our findings primarily concern social norms amongst middle-aged rather than older-aged Malawians and may not generalise to countries with markedly different population age-structures. Second, our findings under-represent the views of those who declined HIVST, many of whom may have been older. Third, we conducted qualitative studies within the context of broader cluster-randomised trials that primarily delivered door-to-door HIVST. Therefore, preferences on HIVST may have included more general preferences for door-to-door and community-based HIV testing. Fourth, because more women were recruited into the study, and that there was not a male only FGD, the views of men may be under-represented. Lastly, it is possible that a young male data collection team (age < 35 years) may have influenced the quality of data collected especially from women and older participants. However, the experience of the team and support from senior qualitative researchers (ND) helped overcome this challenge.

## Conclusions

Age and gender norms are important drivers of HIV risk perception and HIV testing uptake during midlife-older adulthood. Using the life-course approach we highlight how age and gender norms contribute to poor uptake of conventional HIV testing by middle-aged or older individuals, as well as preferences for future self-testing in Malawi. With changes in HIV epidemiology, the increasing ease of access to ART and new HIV testing options (such as HIVST), there is an urgent need to provide targeted messages and services more appropriate to this age-group in sub-Saharan Africa. These messages need to include information on HIV risk and the importance of condoms later in life, as well as education on the benefits of ART including that PLHIV on ART who maintain viral suppression will not transmit HIV to their partners.

Despite concerns that HIV testing in facilities would be viewed as disreputable and undermine the current and future social status of midlife-older adults, HIVST appeared to provide a safe and acceptable alternative for mature adults to test, without challenging social age or gender expectations. While door-to-door, with an extra kit for a partner and support for the self-testing, continues to be preferred by middle-aged and older adults, additional service delivery approaches were considered age-gender appropriate (i.e. clinics for women, discreet community collection points and workplaces for men). Future programmes should consider these preferences as they plan HIV testing services, including HIVST scale-up, among midlife and older adults.

## Supplementary Information

**Additional file 1: Table S1.** Detailed summary of participant quotes from focus group discussions and in-depth interviews across the life course.

## Data Availability

As agreed in the LSHTM, and Malawi College of Medicine and Research and Ethics Committee (COMREC) ethics approval and research protocol, qualitative interview transcripts and the corresponding anonymised NVivo file are only visible to the direct research team, and are not publicly available.

## Abbreviations

CBD: Community-based distributors
COMREC: College of Medicine and Research and Ethics Committee
FGD: Focus group discussions
HIVST: HIV self-testing
IDI: In-depth Interviews
LSHTM: London School of Hygiene and Tropical Medicine
PLHIV: People living with HIV
Q: Quote
STAR: Self-Testing Africa
