# Supplementary material for: ‘Too old to test?’: A life course approach to HIV-related risk and self-testing among midlife-older adults in Malawi
Source: BMC Public Health. 2021 Apr 3;21:650. doi: 10.1186/s12889-021-10573-7 (PMC8019342; doi:10.1186/s12889-021-10573-7)
Supplement: Supplementary file 1 — Additional file 1: Table S1. Detailed summary of participant quotes from focus group discussions and in-depth interviews across the life course. [file 12889_2021_10573_MOESM1_ESM.docx]

**Table S1. Detailed summary of participant quotes from focus group discussions and in-depth interviews across the life course**

| **Key themes** | **#** | **Participant quote** | **Participant characteristics** |
| --- | --- | --- | --- |
| Defining age and midlife-older adulthood | | | |
| Age as chronological | 1a* | [A old person] is over 35 years. A person who is 34 years and below is not [old]…[Because] when you can see that one is 35 years may also have a family. 20 years is not married…[and in a] youthful stage… | Community resident, 35-49 years, urban, Focus group discussion (FGD) |
|  | 2 | In my community old people…their age range starts from 30 [years] going up. | Community resident, 35-49 years, urban, FGD |
|  | 3 | I think there are various stages of being elderly. Someone with 36 years of age -- we call them elderly. | Community resident, 35-49 years, urban, FGD |
| Age as health | 4 | A youth doesn’t have diseases like elderly people. | Community resident, 35-49 years, urban, FGD |
|  | 5 | The youths have much strength and they do any work they can do, so for us the old ones who are 35 years going up, it’s like the strength decreases as we grow. | Community resident, 35-49 years, urban, FGD |
| Age as wisdom | 6 | Because he is older he [is] able to advise children on what to do. | Community resident, 35-49 years, urban, FGD |
|  | 7 | A person can be older but if he has nothing then he is a kid…That is why we say wisdom nowadays is the wisdom that uses money | Community resident, 35-49 years, urban, FGD |
|  | 8 | *[Interviewer: Who are usually in positions such as members of parliament or chairmen?]*  [Those] positions do not look at whether this person is young or old, but the experience…and how that person is. Some can be old but they can fail to look after the village. So even a youth can hold a big position in the village. | Community resident, 50+ years, urban, FGD |
| Age as life events | 1b* | [A old person] is over 35 years. A person who is 34 years and below is not [old]…[Because] when you can see that one is 35 years may also have a family. 20 years is not married…[and in a] youthful stage… | Community resident, 35-49 years, urban, FGD |
|  | 9 | I have started a motherly life, so I can now be doing this and that; all that I was doing in the past was childish | Community resident, 35-49 years, urban, FGD |
|  | 10 | …In the past a person who is 40 years was able to give birth to maybe 15 children. | Community resident, 35-49 years, urban, FGD |
|  | 11 | …it is not wise…your children giving birth and [that] you are also giving birth. | Community resident, 35-49 years, urban, FGD |
|  | 12 | If you find a [young girl] you find that in her childish ways she gets pregnant…and delivers a child. | Community resident, 50+ years, urban, FGD |
| Age as responsibility | 13 | The elderly person is the one who has the responsibility in the family because for example at 40 years that means he has a lot of children…the one who is working is the same elderly person…because there are not many children who have been educated and they are working | Community resident, 35-49 years, urban, FGD |
|  | 14 | The one who takes up responsibilities is an old woman. | Community resident, 35-49 years, urban, FGD |
| Age as behaviour | 15 | If your behaviour is not good…even a child thinks you are also a child. While if a little child gives himself respect, some people also respect him as if he is older. | Community resident, 35-49 years, urban, FGD |
|  | 16 | The one who is looked at as an old person, is the one who follows the advice that he has been given, because when an old person is told something he follows the rules. The one whom we consider as a young person, is the one who doesn’t follow the advice that people give him. | Community resident, 35-49 years, urban, FGD |
|  | 17 | We know a young person [by] their behaviour…it’s like a prodigal life. They drink a lot, smoke chamba, fornication is becoming rampant in the young ones. | Community resident, 35-49 years, urban, FGD |
| Gender roles and norms | | | |
| Head of household | 18 | In terms of my home, I always make sure that whenever there is a problem in the family such as shortage of food or insufficient clothing, I should solve it. I may not deal with it wholly, but at least I try my level best. Further to that, I also participate in the development endeavors of our community that needs the participation of men…The obligations of a man in a family [is] to ensure that the household has enough food, buying clothes for the children but also living in an appropriate environment. | Community resident, 35-49 years, rural, IDI |
|  | 19 | The first thing is to find money, and the second thing is when you find that money you should build a house and buy home necessities. Even people will appreciate that “yes, this man is doing his job as a man”. But if you can’t find money, there is food scarcity [for] your family because there is no money. There might be poverty at your family. | Community resident, 50+ years, rural, FGD |
| Taking care of children and household | 20 | There are many responsibilities. She has to look for food and also cultivating the fields. Even if she is not the one tilling the ground, she must play a supervisory role to the work in the field…She must make sure her household members are eating a well-balanced diet from all the six groups. | Community resident, <35 years, rural, in-depth interview (IDI) |
|  | 21 | [Being a woman, it might be dressing and the way she talks…[When it comes to taking care of her own health or the family’s health], she should have a hardworking spirit at work or at business and be independent. | Community resident, 35-49 years, rural, IDI |
| Fidelity and trust | 22 | [Being a woman means] behaviour as well, as a woman doesn’t need to go around with other men apart from her own husband. | Community resident, 35-49 years, rural, IDI |
|  | 23 | Being a man, it means you are a man. I can say that, you would have sexual feelings to other women, but it just takes the person’s mind [to decide] whether to go for it or not, [and not just thinking] that you are a man, [who] can do anything. | Community resident, 50+ years, rural, FGD |
| Social expectations and standing | | | |
| HIV is a ‘disease of youth’ | 24 | How could an elderly person like this be found with a disease like this? It should have happened to the youth because they are the ones who ‘run faster’ (are more active sexually) | Community resident, 35-49 years, urban, IDI |
|  | 25 | [Older people] think there is no reason to go for testing because in their time there was no HIV. HIV is a disease for people who were born after the year 1985. They think the disease is not part of them as they were born and grew up before the disease was discovered. | Community-based distributor (CBD), 35-49 years, urban, FGD |
|  | 26 | We older people know that this disease is very dangerous - maybe we know that more than the youths do. We have learnt that message through examples, looking after people who have died because of ‘running around’. We are really afraid of it. We have responsibilities and if we go, we know the care in our homes will be decreased. We tell the children ‘you have to be careful because you will contract diseases’. But the youth are not afraid of it. | Community resident, 35-49 years, urban, FGD |
|  | 27 | When an old person is looking at a young person, he thinks that a young person has [HIV] in his body. But when a young person is looking at an old person he is 100% sure that this old person does not have any [HIV] in his body | Community resident, 35-49 years, urban, FGD |
|  | 28 | Most people think AIDS is a disease for the youth, because old people are the ones who give advice. If they are giving advice to the youth and then they should also contract the virus, it becomes surprising. It makes people ask a lot of questions. | Community resident, 35-49 years, urban, FGD |
| HIV-related risk perceptions | | | |
| Infidelity and trust | 29 | No I don’t have concerns, even though I am not in marriage but I have a partner and I have trust in her. | Community resident, <35 years, rural, IDI |
|  | 30 | You can say this is my wife and we are loving each other without knowing that you are thinking differently. | Community resident, 50+ years, rural, IDI |
|  | 31 | *[Interviewer: You had any perception of risk of HIV then before found positive with self-testing?]* Yes, because my husband had a relationship with a woman who was HIV positive and she was on ARVs…My husband doesn’t stop his immoral behaviour. | Community resident, 35-49 years, rural, IDI |
|  | 32 | I had the intention to go for testing after noting the results of my husband [his results showed he was HIV positive]. So the results prompted me to have the desire to test as well. | Community resident, 35-49 years, rural, IDI |
| Social acceptability of HIV testing | | | |
| Social and self-stigma | 33 | We explain to them that one can contract the virus through different ways. It might be that you helped a certain person, or maybe you used something sharp, and from nowhere you can easily contract the virus. Because of that, they say ‘I think that you are explaining well’ and you will find that they get tested. | Community-based distributor (CBD), <35 years, urban, FGD |
|  | 34 | …I always have fear with barbershops that cant we get HIV? I just think that because everyone use the same [shaving] machine. | Community resident, 50+ years, rural, FGD |
|  | 35 | …I used bath soaps, so maybe through that I can have a concern [HIV risk]. | Community resident, 50+ years, rural, IDI |
|  | 36 | If you go for HIV-testing people say you doubt yourself. They talk a lot saying there is something making you go for testing. They don’t look at it as if it’s just your decision, or it is because you listened to the counselling, or that you wish yourself a better future. They think that maybe you have been sleeping around or maybe you are getting sick. | Community resident, 35-49 years, urban, FGD |
|  | 37 | [Testing] is done quickly when you go to the hospital, and it is better as there are no people there, and everything ends in the room between yourself and the doctor. While at home, you do the testing in front of a lot of children. When a child is there he may be saying ‘counsellors came and they have conducted a test on my mother’. Then somebody could ask ‘what were they testing her for?’, and the child could exclaim ‘AIDS!‘. So you see, that means my neighbours will know that I did the testing with the counsellors. | Community resident, 35-49 years, urban, FGD |
|  | 38 | Some older people ask us what will happen if they are found with the virus - will they receive the drugs right here at home or from the hospital? They say some people feel ashamed to go to the hospital and receive drugs, as there will be a queue for such things. | CBD, <35 years, urban, FGD |
|  | 39 | If an old person has been found with the virus, people tend to wonder saying “aah how come?” because it’s like a young person is the one who is very active in sexual activities. So how has this old man contracted the virus? We look at those old people who contract HIV as if they lack wisdom. | Community resident, 35-49 years, urban, FGD |
| Perceptions and experiences self-testing | | | |
| Disadvantages of HIV testing | 40 | The issue of transport cannot be ruled out because the money to be used for transport could as well be used in the home for other basic needs as such I would rather not go and use the money feed the family. | Community resident, 35-49 years, rural, IDI |
| Recommend self-testing | 41 | *[Interviewer: Would you recommend self-test to your friends or family?]* Very much. If an opportunity emerges that you want to test them, or they want to test themselves and I am around, I would definitely encourage them to use the apparatus because it is very good | Community resident, 35-49 years, rural, IDI |
|  | 42 | Yes, I would recommend because the procedure is simple. | Community resident, <35 years, rural, FGD |
| Benefits of reactive self-test | 43 | Aah, I don’t see any problems. I think there are only benefits because some people are not comfortable to go to the health facility for testing. So it is easier for them to use this method and know their status…Well, [when testing HIV-positive with self-test], I just accepted and admitted it…If I live in denial and be anxious it won’t solve anything. I had to accept and follow the counselling | Community resident, <35 years, rural, FGD |
|  | 44 | The test kit is a very good thing because you are able to read the results yourself instantly. I believed the [positive] results….There is benefit because it [self-testing] will bring trust and love to each other. | Community resident, 35-49 years, rural, IDI |
| Ease of use and time saving | 45 | It was very simple to self-test, I just followed the instructions and managed to test myself….I would recommend self-testing because we save time instead of going to HTC [HIV testing and counselling] we do it ourselves at home. When you think about time and cost, is better to use self-testing because you will do it while at home. Self-test and you don’t waste your time, while at HTC you need to travel and spend money for transport and you will be tested by the doctor. | Community resident, <35 years, rural, IDI |
|  | 46 | [Self-testing] at home - you can do that within fifteen minutes while you are doing other things at home, while testing at a facility it can take you over an hour. | Community resident, <35 years, rural, IDI |
| Preferences for support | | | |
| Support during self-testing | 47 | The counselling regarding the kit itself would be to highlight how the apparatus works or how we can use it. After knowing how it works, then we would be able to use it. The only assistance I would want is advice regarding how to properly use the kit. | Community resident, 35-49 years, rural, IDI |
|  | 48 | The counsellor should provide counselling only, not monitoring the person. | Community resident, 35-49 years, rural, IDI |
|  | 49 | [Without guidance and supervision] it [would] be difficult because you don’t even know how to open the pack. For other people they can be easily to understand, while others it may be difficult for them, so to others might bring confusion. | Community resident, 50+ years, rural, FGD |
|  | 50 | Old people prefer different things. Those who have reached 45 to 70 years are the ones who test in our presence so that we should help them in reading the results, and so you can explain the instructions to them properly. But people who are 28 to 40 years like to test by themselves because they know that may be their behaviour was not right at a certain time, and they know it wouldn’t be a problem to go to the hospital themselves. | CBD, 35-49 years, urban, FGD |
| Preferences kit distribution of self-test kits | | | |
| Expectations of CBDs | 51 | Old people are stubborn to hear any advice from children. They don’t believe these children. They look at themselves as old people who have more wisdom. So, if a young counsellor goes to such a person, will they listen to him? | Community resident, 35-49 years, urban, FGD |
|  | 52 | *[Interviewer: Who should distribute self-test kits in terms of age and sex?]* Anyone, as long as the person is trustworthy | Community resident, 35-49 years, urban, IDI |
|  | 53 | …Maybe your child will be conducting a test on me. I am an old person. | Community resident, 50+ years, urban, FGD |
| Home-based distribution | 54 | The best distribution is like what happened last time, because many people were received. So if they can continue to distribute on the same way [door-to-door], I believe many people will know their HIV status. I think if they pass through the Village head man, people will not go but they should reach them through door to door. | Community resident, 50+ years, rural, FGD |
|  | 55 | At our village headman’s residence because we have [outreach family planning clinic] every month, so that place would be easy for everyone to get the self-testing kit. | Community resident, <35 years, rural, IDI |
| Facility-based distribution | 56 | [HIV self-tests should be distributed] in all our nearest health centres like Zingwangwa, Mpemba and Pensulo. | Community resident, <35 years, rural, IDI |
|  | 57 | [Self-tests] should be at the hospital, may be at K1.000.00 as a price. | Community resident, 35-49 years, rural, IDI |
| Other: Community collection points, workplace, bus depots | 58 | Create a collection point [for men] at the same chief’s compound. | Community resident, 35-49 years, rural, IDI |
|  | 59 | The place could be somewhere closer to us so that we do not need to spend money on transport in order to reach the place. Finding a good place which we feel that this place is close to us and that even the people around there can easily access it. | Community resident. 35-49 years, rural, IDI |
|  | 60 | It needs to be distributed especially in the companies, bus depot and other areas that are largely men available. | Community resident, <35 years, rural, IDI |
| Pre- and post-test support | | | |
| Phone or in-person | 61 | Through phone can be the best way, because through a letter the person might be illiterate so that would be difficult to understand. | Community resident, 50+ years, rural, FGD |
|  | 62 | I don’t think there should be anything to worry about because the message can be delivered anyhow [by phone or in-person], even at a public rally. There you are not targeting one person but a group of people which has gathered there. | Community resident, <35 years, rural, IDI |
|  | 63 | Some people do not have access to phones, so the best way is face to face. | Community resident, <35 years, rural, IDI |
| Partner self-testing | | | |
| Family and couples counselling | 64 | The best counselling should be provided as a couple, because they will remind each other if one has forgotten. | Community resident, 50+ years, rural, IDI |
|  | 65 | Counselling given to a family as a whole is good because it gives an opportunity for everyone to hear for himself… Especially [for] me and my wife | Community resident, 35-49 years, rural, IDI |
| Give kit to partner | 66 | Yes, I would be very glad because me and my wife are one. So if that could be the arrangement I believe she would be very glad to, because from the very beginning she was the one who was encouraging me to go for blood testing. With this kit, my wife would also be able to test herself. | Community resident, 35-49 years, rural, IDI |
|  | 67 | I found myself to be HIV positive together with my husband. [Before] we had plans to go for testing, so we took self-testing together as an advantage to us, [and] we accepted the results…There is benefit because it [self-testing] will bring trust and love to each other. | Community resident, 35-49 years, rural, IDI |
| Perceived benefits of treatment | | | |
| Fatalism | 68 | Some people say they are already dead when they test HIV positive, instead of start to receive ARVs. | Community resident, 50+ years, rural, IDI |
|  | 69 | Some older people say ‘I have already grown up – what is remaining here is just dying. Why should I go to test? Even if they will mend me, what will that do for me?’ | Community resident, 50+ years, urban, FGD |
| Condom use and abstinence | 70 | Since that incident happened [both diagnosed with HIV], the community health worker came and gave us condoms. That’s what we are using now. We are using condoms, apart from that we usually having sex once per week or two weeks. | Community resident, 35-49 years, rural, IDI |
|  | 71 | Yes, we use [condoms]…We will continue. As for this unborn baby, [I am protecting it through] the treatment I am receiving. | Community resident, <35 years, rural, IDI |
| Linkage to care | | | |
| Accompaniment or assistance | 72 | Relatives can help you to link with support and care services by confirming to doctors that indeed you are HIV positive. | Community resident, 50+ years, rural, IDI |
|  | 73 | The person can be assisted if there is a health worker nearby. | Community resident, <35 years, rural, IDI |
| Referrals, including letters or slips | 74 | I will need to get a letter [referral slip] from your organization to show at the hospital, that can be better. | Community resident, 35-49 years, rural, IDI |
| Incentives | 75 | After counselling the person advised them to go to the service centre to receive medication, maize flour, cooking oil and other things. | Community resident, 50+ years, rural, FGD |
| Social harms | | | |
| No concerns, just benefits | 76 | There would be no risks apart from benefits for everyone who will know his/her HIV status. | Community resident, 50+ years, rural, FGD |
|  | 77 | No that cannot happen because whoever go for self-testing that means has made a decision. | Community resident, 35-49 years, rural IDI |
|  | 78 | Aah, I don’t see any problems. I think there are only benefits…There isn’t any threat. | Community resident, <35 years, rural, IDI |
| Re-use of self-test kits | 79 | Those self-testing kits needs to be taken care of and kept on safe place to avoid reuse of the kits. | Community resident, <35 years, rural, IDI |
| Not disclosing reactive results | 80 | The problem might be if you found HIV positive and haven’t disclosed to health workers that might be a problem. | Community resident, <35 years, rural, IDI |
| Harming self or others | 81 | To me what I envisage as a threat to this program mainly relates to when people know their results. Some people if found positive may take it as the end of their life and decide to infect as many people as possible so that they are not alone with the virus. The only way to prevent that is to ensure that a person is properly counselled before the testing and that he understands that whatever results that may come out, should not cause him to get confused and start misbehaving. | Community resident, 35-49 years, rural, FGD |
